# Acid red 18 removal from aqueous solution by nanocrystalline granular ferric hydroxide (GFH); optimization by response surface methodology & genetic-algorithm

**DOI:** 10.1038/s41598-022-08769-x

**Published:** 2022-03-19

**Authors:** Farshad Hamidi, Mohammad Hadi Dehghani, Mahboobeh Kasraee, Mehdi Salari, Leila Shiri, Amir Hossein Mahvi

**Affiliations:** 1 Department of Environmental Health Engineering, School of Public Health, Tehran University of Medical Sciences, Tehran, Iran; 2 Institute for Environmental Research, Center for Solid Waste Research, Tehran University of Medical Sciences, Tehran, Iran; 3 Department of Environmental Health Engineering, School of Public Health, Hamadan University of Medical Health, Hamadan, Iran

**Keywords:** Environmental chemistry, Environmental sciences

## Abstract

The need for fresh water is more than before by population growth, and industrial development have affected the quality of water supplies, one of the important reason for water contamination is synthetic dyes and their extensive use in industries. Adsorption has been considered as a common methods for dye removal from waters. In this study, Acid Red18 removal in batch mode by using Granular Ferric Hydroxide (GFH) was investigated. The GFH characterized by XRD, FESEM and FTIR analysis. Experiments were designed using RSM-CCD method. The maximum removal efficiency was obtained 78.59% at pH = 5, GFH dosage = 2 g/l, AR18 concentration = 77.5 mg/l and 85 min of contact time. Optimization with RSM and Genetic Algorithm carried out and is similar together. The non-linear adsorption Isotherm and kinetic fitted with Freundlich (R2 = 0.978) and pseudo-second-order (R2 = 0.989) models, respectively. Thermodynamic studies showed that the AR18 adsorption is endothermic process and GFH nature was found spontaneous.

## Introduction

Water pollution and its related problems have already generating considerable interest, and Trying to deal with this pollutant has become a priority nowadays^1^. industrial development and their following wastewater production have raised many problems in aquatic sources^2^. Many industries such as textile, paper, plastic, leather, cosmetics, food products, and pharmaceuticals^3^ widely use synthetic dyes and generate a highly colored wastewater^4,5^. One of the main causes of water pollution is synthetic dyes^6^ and consist of several different groups including of reactive dyes, direct dyes, basic dyes, azo dyes and other groups^7^. One of the main section of synthetic dyes are azo dyes which formed by an azo group (N=N) that can have carcinogenic and mutagenic impact and aesthetic problems^8–10^. Some dyes are toxic, non-degradable and affect human, animal and environmental health^6^. It is visible even in concentrations less than 1 ppm in water^3^. However, azo dyes even at low concentrations can lead to a disorder in the photosynthesis process and cause some problem in oxygen solubility in water sources^11,12^. Acid Red 18 is categorized as an azo dye^13^. About 10–15% of the produced colors in dying process discharge to the sewage^14–17^. Due to environmental pollutions which are the result of releasing untreated colored-wastewater to water sources, the importance of its removal from water solutions has increased. That is why the removal of such dyes from industrial effluents is extremely important; Conventional treatment methods for removing dyes from wastewater are less effective^18^. Methods physical and chemical like adsorption, photocatalytic process, coagulation and flocculation, biological treatments, advanced oxidation precipitation^19^ electrochemical technique^18^ have been applied for colored wastewater treatment^20^. Among all prenominated methods, adsorption is a more used and an economical method for contaminated waters treatment^21^; and ease operation, and low energy consumption^22^. This method It has attracted a lot of attention because of high efficiency, environmentally and higher quality effluent^1,8^. Researchers have used different adsorbent for this aim. Granular Ferric Hydroxide (GFH) is mainly formed by Akaganeite, a poorly crystallized β-FeOOH and was used as an adsorbent for effective removal of arsenic^23^ NOM^24^ fluoride^25^ and bromate^26^. High removal efficiency and relatively low cost have been observed in the group of iron compounds (oxides and hydroxides, including amorphous hydrated ferric oxide (FeOOH) and goethite^27^. In a few researches, application of Moringa seeds and coagulation by alum for treatment of textile wastewaters have shown promising results as well^28^. In present study application of GFH in removal of Acid Red 18 was assessed. In most studies, the efficiency of this adsorbent in the removal of heavy metals, especially arsenic and anions, has been studied, and there is few studies on its performance in relation to organic compounds and the aim of this study was to investigate the structure of granules GFH and optimize the removal of acid red 18 by RSM method and genetic algorithm.

## Material and method

### Materials

AR18 dye was provided from Alvan Sabet Company, Hamedan, Iran. Also Granular Ferric Hydroxide from GEH Wasserchemie GmbH & Co. KG Company. All other chemicals were purchased from Merck (Darmstadt, Germany).

### Adsorbent characterization

To determine surface morphology of GFH, Field Emission Scanning Electron Microscope (FESEM) was used. Phase identification of a crystalline material done with X-ray diffraction (XRD) and The Functional groups of the adsorbent were studied by a Fourier transformed infrared (FTIR) spectrometer.

### Analytical measurements

**Figure 1:**
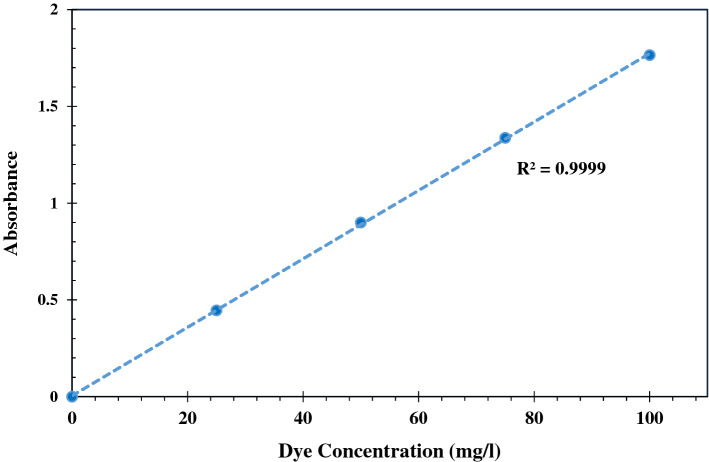
AR18 calibration curve.

**Figure 2:**
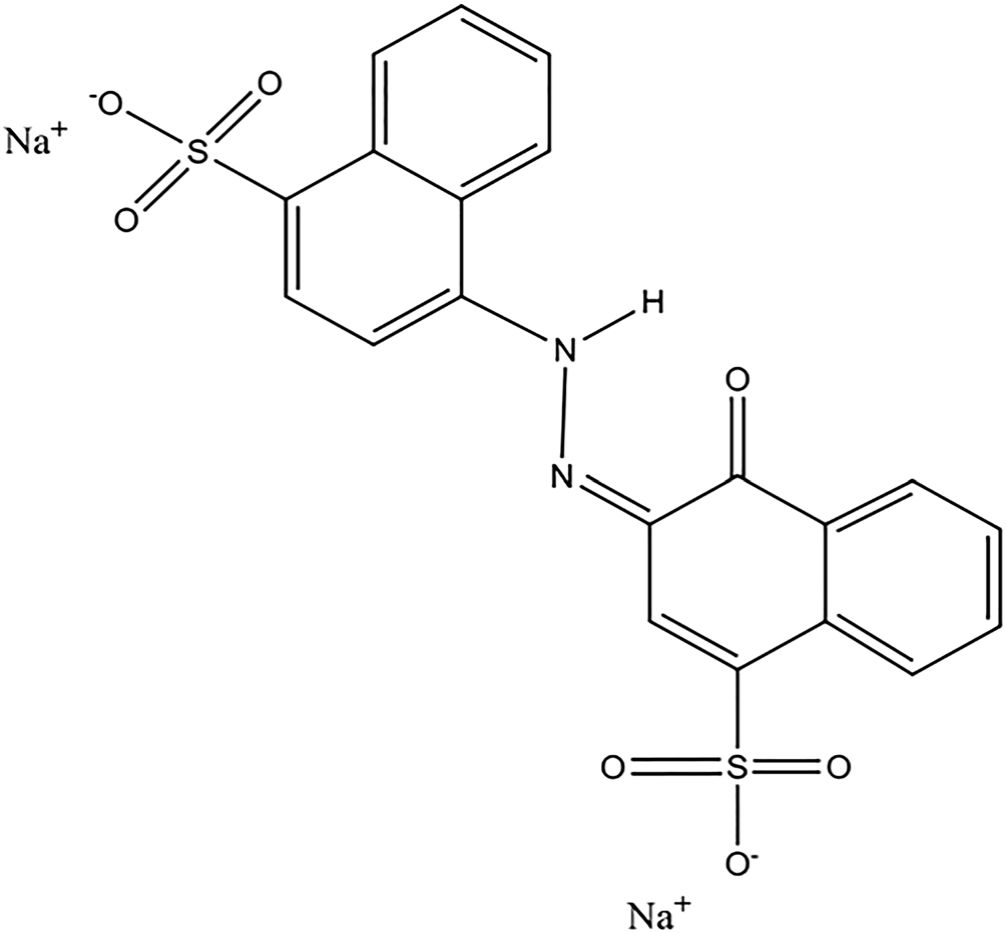
Acid red 18 molecular structure.

AR18 dye stock solution (500 mg/l) was made by dissolving AR18 powder in distilled water and desired concentrations of AR18 were prepared. The initial and residual concentration of dye solution were determined by UV–visible spectrophotometer (Perkin Elmer Lambda 25) at λ = 507 nm. The calibration curve was plotted by 4 concentrations of dye between 0 and 100 mg/l which is shown in Fig. 1. Also molecular structure of Acid red 18 anionic dye is drawn in Fig. 2. As can be seen in Table 1, due to the presence of moisture in the structure of this adsorbent, it was placed in an oven (memmert) for 24 h in order to prepare for the adsorption process, and then cooled in a desiccator.

**Table 1:** GFH properties.

Property	Unit	Value
Saturation	%	43–48
Porosity	%	72–77
pH	–	7.5–8.2
Specific surface	m^2^/g	280
Effective size	mm	0.32–1
Uniformity coefficient	–	About 3

### Batch adsorption studies

Dye adsorption studies were performed in 100 ml glass Erlenmeyer flasks with 25 ml of dye solution. Desirable pH was adjusted by 0.1 N NaOH and HCL by Kent EIL7020 pH meter, a certain amount of adsorbent weighted and added to the dye solution and then samples were shaken at room temperature at 200 rpm. After that flask contents were centrifuged in 4500 rpm for 5 min in Universal Premium PIT320 model and the residual dye concentration read by spectrophotometer Perkin-Elmer lambda25. The amount of adsorbed dye onto the adsorbent surface was calculated by using the mass balance equation:

$${\mathrm{q}}_{\mathrm{e}}=\left({\mathrm{C}}_{0}-{\mathrm{C}}_{\mathrm{e}}\right)\frac{\mathrm{v}}{\mathrm{m}}$$

C0 is the initial concentration of dye (mg/l), C*e* is the residual concentration of dye (mg/l), M is the mass of adsorbents (g), V is the volume of AR18 solution (l).

Furthermore, dye removal efficiency was obtained from:

$${y}_{\%}=\frac{{c}_{0-{\mathrm{C}}_{\mathrm{e}}}}{{c}_{0}}\times 100$$

where C0 is the initial concentration of dye (mg/l), C*e* is the residual concentration of dye (mg/l).

Isotherm studies were carried out at different dye concentration, and the kinetic was determined at different contact time. Thermodynamic studies were performed at various temperatures for understand the nature of the process.

### Experimental design

Design-Expert11 (Stat.Ease.Inc. Minneapolis, USA) software with Response Surface Methodology (RSM) package applied to design experimental runs and optimum conditions and the impact of various independent variables (pH, initial concentration, contact time, adsorbent dosage). Dye adsorption was evaluated by using central composite design (CCD) which can minimize the numbers of total experiments. Likewise, to determine a total number of runs the following equation is presented: N = 2^k^ + 2k + C_0_.

In which K is the number of experimental variables, 2^k^ is the cubic runs, 2k is the axial runs, and C_0_ is the center point's runs^29^. CCD matrix and coded values of variables is shown in Tables 2 and 3.

**Table 2:** Independent variables and the levels of each variable.

Variable	Symbol	− α	− 1	0	+ 1	+ α
pH	X_1_	3	5	7	9	11
Adsorbent dosage (g/l)	X_2_	0.5	1	1.5	2	2.5
Contact time (min)	X_3_	10	35	60	85	110
Acid red 18 concentration (mg/l)	X_4_	10	32.5	55	77.5	100

**Table 3:** CCD design for AR18 removal.

Std	Run	A: pH	B: Dose (g/l)	C: Time (min)	D: AR18 (mg/l)	Ce (mg/l)	RE (%)	Predicted value (%)
22	1	7	1.5	110	55	36.8	33	34.26
21	2	7	1.5	10	55	49.87	9.32	10.62
1	3	5	1	35	32.5	25.1	22.7	22.60
6	4	9	1	85	32.5	30.8	5.23	5.98
29	5	7	1.5	60	55	46.52	15.42	16.56
25	6	7	1.5	60	55	44.8	18.6	16.56
30	7	7	1.5	60	55	44.9	18.3	16.56
23	8	7	1.5	60	10	6.5	34.9	34.12
14	9	9	1	85	77.5	58.5	24.5	21.99
7	10	5	2	85	32.5	11.4	65	62.72
24	11	7	1.5	60	100	40	60	63.34
12	12	9	2	35	77.5	41.9	46	44.39
27	13	7	1.5	60	55	46.6	15.3	16.56
28	14	7	1.5	60	55	46.58	15.31	16.56
11	15	5	2	35	77.5	31.48	59.43	57.42
8	16	9	2	85	32.5	26.8	17.55	18.29
17	17	3	1.5	60	55	20.87	62	63.63
2	18	9	1	35	32.5	31.3	3.69	3.51
18	19	11	1.5	60	55	48.3	12.18	13.10
15	20	5	2	85	77.5	15.72	79.71	78.59
13	21	5	1	85	77.5	41.6	46.31	46.50
9	22	5	1	35	77.5	58.1	25	22.96
5	23	5	1	85	32.5	18.47	43.17	43.48
26	24	7	1.5	60	55	45.96	16.44	16.56
19	25	7	0.5	60	55	51.8	5.81	5.80
10	26	9	1	35	77.5	65.22	15.84	16.86
4	27	9	2	35	32.5	26.12	19.63	18.18
20	28	7	2.5	60	55	27.5	50	52.56
3	29	5	2	35	32.5	18.52	43	44.21
16	30	9	2	85	77.5	40.06	48.3	47.14

This approach was suggested 30 trials. The following are some of the benefits of this design:

(i) It proves to be a two-level or fractional factorial design extension. (ii) Determine the nonlinearity of responses in the data set. (iii) Assists in estimating curvature in continuous answers received. (iv) In a minimum experimental run, the most information is obtained.

### Isotherm studies

Adsorption isotherms provide quantitative information on an adsorbent's adsorption capacity and behavior with the adsorbate^19^. Experimental data were analyzed and fitted to Freundlich, Langmuir, and Temkin isotherm models to achieve this goal.

#### Langmuir isotherm

The Langmuir isotherm is used to describe monolayer adsorption on a homogenous adsorbent surface^30^. The following equations reflect its nonlinear model:

$$ q_{e} = \frac{{q_{m} K_{L} C_{e} }}{{1 + K_{L} C_{e} }} $$

where q_e_ is the amount of AR18 adsorbed per gram of GFH, C_e_ is the dye concentration at equilibrium (mg/l), and q_max_ is the maximum amount of Acid red18 adsorbed. The Langmuir equilibrium constant in l/mg is denoted by K_L_. Non-linear models were used to estimate the parameters.

#### Freundlich isotherm

Adsorption is not monolayer in the Freundlich isotherm, which is formulated for equilibrium on inhomogeneous surfaces^31^.

The nonlinear version of the Freundlich isotherm model is as follows:

$${q}_{e}={K}_{f}{C}_{e}^\frac{1}{n}$$

Adsorption capacity is represented by K_F_ (l/mg), while adsorption intensity is represented by 1/n. The nonlinear plot of qe versus Ce and the linear plot of log qe vs. log Ce were utilized to calculate K_F_ and n.

#### Temkin isotherm

A heterogeneous surface with adsorption sites with the same bond energy is studied in the Temkin isotherm model. A linear reduction in adsorption heat occurs in this isotherm due to the contact effects of the adsorbent and adsorbate on each other^31^. The following equation expresses the non-linear Temkin models:

$$ {\text{q}}_{{\text{e}}} = \frac{RT}{{b_{{}} }}{\text{lnK}}_{T} {\text{C}}_{{\text{e}}} { } $$

B is the isotherm constant (J/mol). The maximal bond energy (l/mg), the gas constant (8.314 J/mol K), the temperature in degrees Kelvin, and the heat of adsorption (J/mol) are all represented by the letters K_T_, R, T, and b.

### Kinetic studies

The pace and mechanism of the reaction are revealed by a kinetic examination of the adsorption process^32^. Ion adsorption from solution has been studied using a variety of kinetic models. The adsorption kinetic of AR18 onto GFH was studied using pseudo-first order, pseudo-second order, and intraparticle diffusion kinetic models. These three models' nonlinear forms are written as:

$$ {\text{Nonlinear Pseudo first order kinetic equation:}}\quad Q_{t} = Q_{e } \left( {1 - e^{{ - k_{1} t}} } \right) $$

$$ {\text{Nonlinear}}\;{\text{Pseudo}}\;{\text{second}}\;{\text{order}}\;{\text{kinetic}}\;{\text{equation:}}\quad Q_{t} = \frac{{k_{2} Q_{e}^{2} t}}{{1 + k_{2} Q_{e} t}} $$

$$ {\text{Intraparticle diffusion:}}\quad {\text{q}}_{{\text{t}}} = {\text{k}}_{{\text{i}}} {\text{t}}^{0.5} + {\text{C}} $$

where q_e_ (mg/g) is the adsorption capacity at equilibrium and q_t_ (mg/g) is the adsorption capacity at time t. Adsorption rate constants are k_1_ (/min) for pseudo-first-order kinetic, k_2_ (g/mg min) for second-order kinetic, and k_i_ (mg/g min^0.5^) for intraparticle Diffusion kinetic^30^.

### Thermodynamic study

Thermodynamic parameters include changes in the free energy of Gibbs (ΔG), enthalpy (ΔH^o^), and entropy (ΔS). ΔG is free energy change (kJ/mol) can be calculated from the following equation

$$\Delta G=-RT\left(\mathrm{ln}{K}_{c}\right)$$

where R is the universal gas constant (8.314 J/K mol), T is the absolute temperature (K). The distribution adsorption coefficient, Kc, is calculated from the following equation:

$$\frac{{q}_{e}}{{C}_{e}}={kc=\frac{C0-ce }{ce} \frac{\mathrm{v}}{m}}$$

where C_0_ is the initial concentration (mmol/l), Ce is the equilibration concentration after centrifugation (mmol/l), V is the volume of the suspension (l), and m is the mass of adsorbent (g). The adsorption equilibrium constant (K) can be calculated by plotting lnK_d_ versus Ce and extrapolating Ce to 0. The value of the intercept is that of lnK. **ΔH**^**o**^ (J/mol—kJ/mol) and ΔS_0_ (J/mol °K—kJ/mol K) according to Equation are calculated.

$$-\frac{\Delta H^\circ }{RT}\mathrm{ln}{ K}_{c}=\frac{\Delta S^\circ }{R}$$

where **ΔH**^**0**^ is the isosteric enthalpy change, ΔS is the entropy change, where **ΔH**^**0**^ and ΔS^0^ were obtained from the slope and intercept of the linear plot of lnK_d_ against 1/T^32^. The number of tests for isotherm, kinetic and thermodynamic studies was selected in range, above which, the q_e_ alter not significantly. In this regard, the number of tests was 8 for each kinetic model, 7 for each isotherm models, and 3 for thermodynamic study based on the temperatures selected.

## Results and discussion

### Granular ferric hydroxide characterization

#### XRD studies

**Figure 3:**
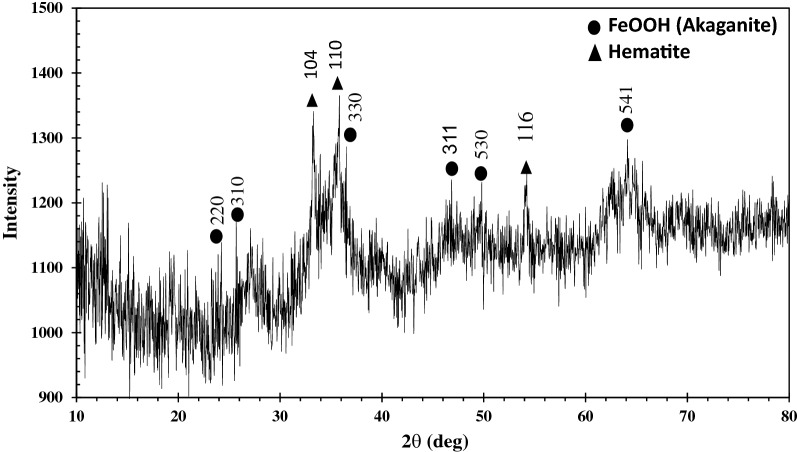
XRD pattern of GFH.

X-ray diffraction of GFH was analyzed in the range of 2θ = 5–80° at 0.02 step size, and the result is shown in Fig. 3.

Akaganeite was found to be the main compound in GFH structure and also there was hematite in lower content. Baseline noise and peak broadening related to akaganeite reveals the poor crystallinity in GFH structure which is in line with previous reports on GFH characteristics that defined β-FeOOH as a poor crystallized similar to mineral akaganeite. akaganeite is a tetragonal mineral formed by a double chain octahedral^23^. On the other hand with existing a low amount of chloride in GFH sample, broadening of peaks in XRD is observable^23^. The peaks at 24.22 (220), 25.68 (310), 33.2 (104), 35.8 (110), 36.42 (330), 46.84 (311), 49.8 (530), 54.15 (116) and 64.11° (541) indexed and matching with JCPDS file no. 34-1266 and 33-0664.

#### FESEM

**Figure 4:**
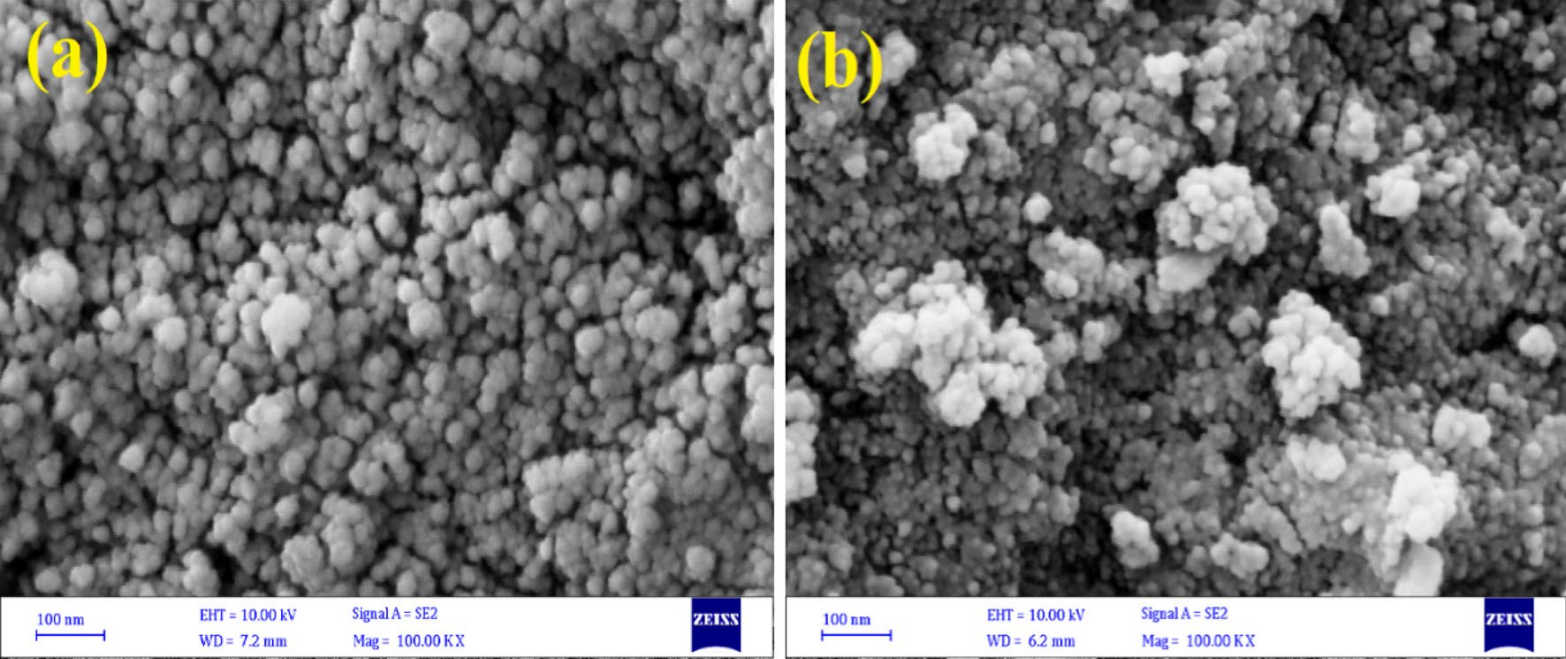
GFH FESEM (**a**). Raw (**b**). After AR18 adsorption.

**Figure 5:**
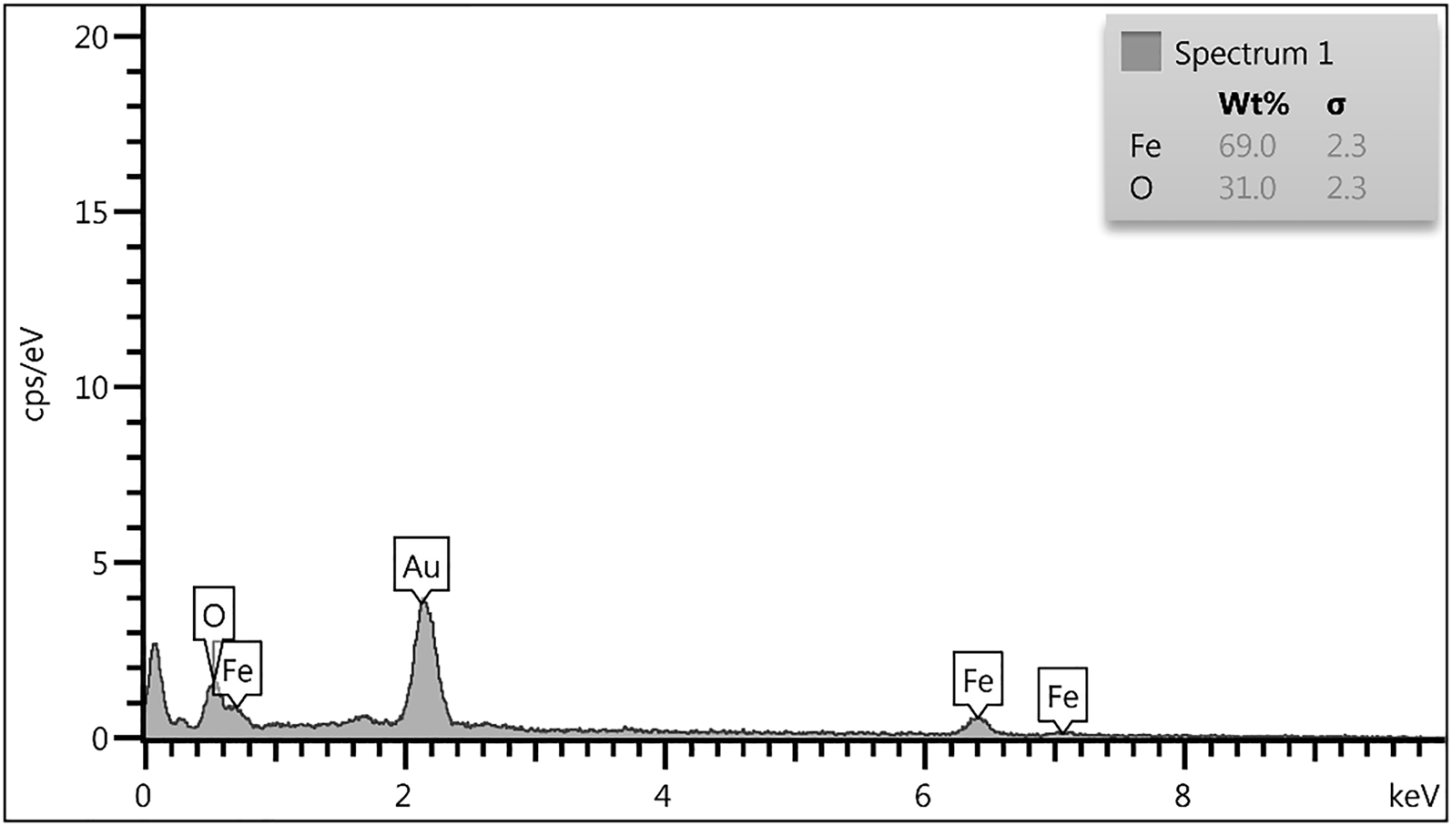
GFH EDS spectrum.

Figure 4a shows the surface morphology of GFH. It displays uniform round edge of GFH particles and also has a porous structure which can provide suitable adsorption sites for dye removal as shown in Fig. 4b these sites are appropriately filled with dye molecules, and sorbent particles have become more agglomerated. EDX analysis (Fig. 5) determined to contain elements in GFH. The result indicates that Fe and O are the two main constituent of GFH with 69 and 31% respectively.

#### FTIR analysis

**Figure 6:**
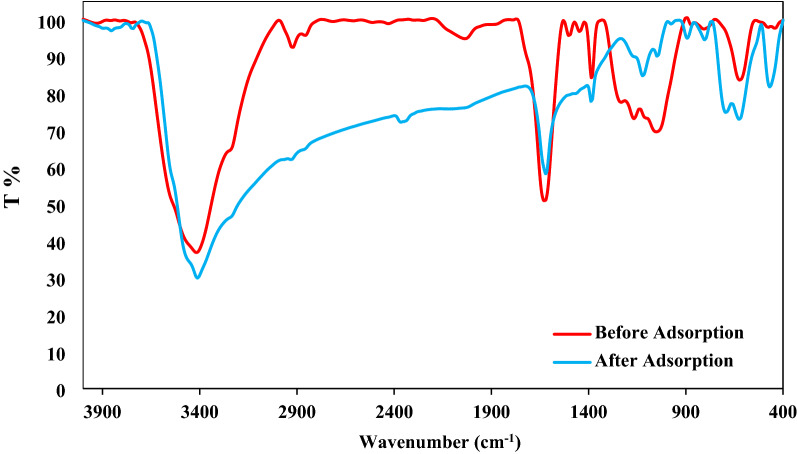
AR18 FTIR before and after of adsorption.

The Fourier transform infrared (FTIR) analysis was used to identify functional groups of GFH sample by PerkinElmer, Spectrum Two. Samples were studied at the range of 400–4000/cm, Fig. 6 shows the FTIR spectra of Granular Ferric Hydroxide before and after adsorption of AR18.

Bands at 2928 and 2858/cm are associated with symmetric and anti-symmetric stretching vibrations of the carbon chain^33^, while the O–H stretching vibration may be related to the bands around 3417 and 3411/cm^33^, also the band at 584/cm can be related to Fe–O stretching vibrations^34^, Gong et al. Indicated that the bands assigned to Fe–O appear between 400 and 700/cm^35^ and a weak band at 1123/cm was observed related to Fe−OH^34^. The bending vibration of adsorbed water and surface hydroxyl and O–H stretching mode was situated at 1631\cm^36^.

### AR18 adsorption

#### Statistical analysis

In the present study, four different statistical models and their fitting with obtained experimental data were investigated. Quadratic model with and lack of fit 0.17 was suggested by software analysis. According to the presented data in a Table 4 Quadratic model with adjusted and predicted R^2^ value 0.989 and 0.972 was the best-fitted model for AR18 removal by GFH.

**Table 4:** Fit Summary models of AR18 removal.

Source	Sequential p-value	Lack of Fit p-value	Adjusted R^2^	Predicted R^2^	
Linear	< 0.0001	< 0.0001	0.7042	0.6522	
2FI	0.4788	< 0.0001	0.7012	0.6437	
Quadratic	**< 0.0001**	**0.1736**	**0.9895**	**0.9727**	**Suggested**
Cubic	0.4978	0.0852	0.9896	0.7713	Aliased

Significant values are in [bold].

#### Effect of experimental parameters on adsorption process

The effect of four different variable on AR18 adsorption were studied. From the Table 5, it can understand that pH, Dose, Initial concentration and time with the F-value 851,729,284,186 respectively had the most to the lowest effect on adsorption process efficiency. Also, the interaction of studied variable investigated in which AC showed the bigger F-value it means that the interaction between pH and time had the most impact on dye removal while BC with the lowest F-value was negligible. Also, the high F-value of the quadratic function of D implies the significant effect on adsorption.

**Table 5:** ANOVA for quadratic model.

Source	Sum of squares	df	Mean square	F-value	p-value	
Model	12,320.63	14	880.05	195.61	< 0.0001	Significant
A-pH	3830.93	1	3830.93	851.52	**< 0.0001**	
B-Dose	3279.75	1	3279.75	729.01	**< 0.0001**	
C-Time	838.27	1	838.27	186.33	**< 0.0001**	
D-AR18	1280.71	1	1280.71	284.67	**< 0.0001**	
AB	48.09	1	48.09	10.69	0.0052	
AC	338.93	1	338.93	75.34	< 0.0001	
AD	168.74	1	168.74	37.51	< 0.0001	
BC	5.62	1	5.62	1.25	0.2814	
BD	165.12	1	165.12	36.70	< 0.0001	
CD	7.05	1	7.05	1.57	0.2298	
A^2^	815.07	1	815.07	181.17	< 0.0001	
B^2^	273.02	1	273.02	60.69	< 0.0001	
C^2^	59.17	1	59.17	13.15	0.0025	
D^2^	1773.58	1	1773.58	394.22	< 0.0001	
Residual	67.48	15	4.50			
Lack of fit	55.83	10	5.58	2.40	**0.1736**	Not significant
Pure error	11.65	5	2.33			
Cor total	12,388.12	29				

Significant values are in [bold].

**Figure 7:**
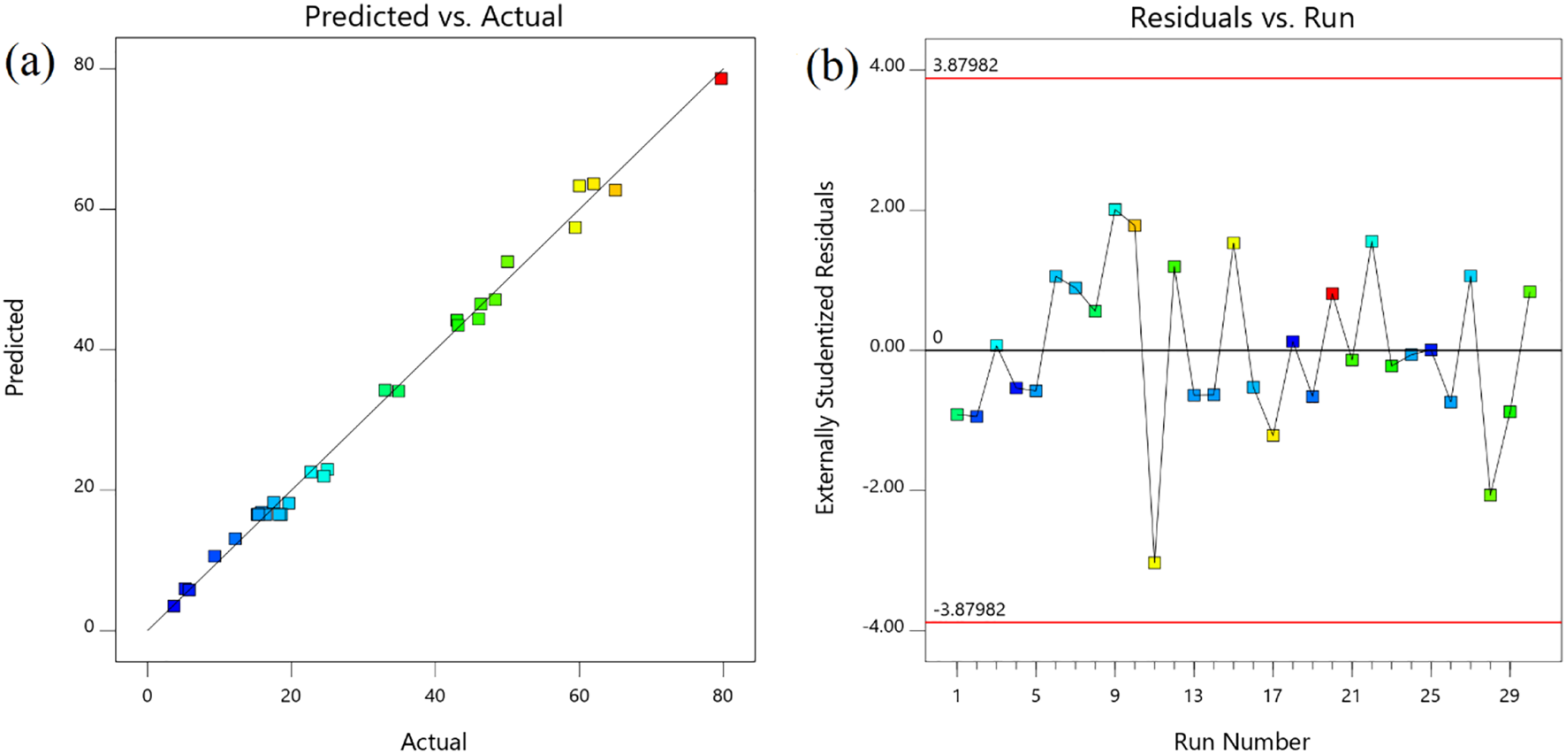
Predicted vs. actual (**a**) and residual vs run (**b**).

Predicted vs actual efficiency comparison is shown in Fig. 7a. As it can be seen, there was a good correlation between them. Also residual of runs was in the range of − 3 to 3, which is indicant of a close value to the predicted one (Fig. 7b).

#### Effect of pH

The negative coefficient of pH was indicative of decreasing removal efficiency with increase in pH; this can be explained that at acidic pH, the higher AR18 uptake will occur. Due to the presence of the large number of positive charges on the adsorbent's surface at lower pHs while on the other hand the AR18 Molecules are negatively charged and this lead to an Electrostatic sorption^2^ The calculated pH_ZPC_ value for GFH was determined 7.5–8 based on previous studies. The sorbent surface charge is more positive at pH < pH_ZPC_ while at pH > pH_ZPC_ the AR18 adsorption shows decrease, and as a consequence, the removal efficiency will be decreased.

#### Effect of initial dye concentration

One of the effective parameters on adsorption process in this study was the initial concentration of AR18. The impact of this parameter was studied. The result showed that with an increase in initial concentration of dye, the efficiency improved. It might be because of more availability of pollutant for adsorbent particles.

#### Effect of adsorbent dosage

As it can be seen adsorbent dosage is the most important parameters effects on AR18 adsorption on GFH. Understandably, GFH provides more surface area for adsorption of dye; besides, it was found that a higher amount of GFH, a higher removal efficiency could be achieved.

#### Effect of contact time

**Figure 8:**
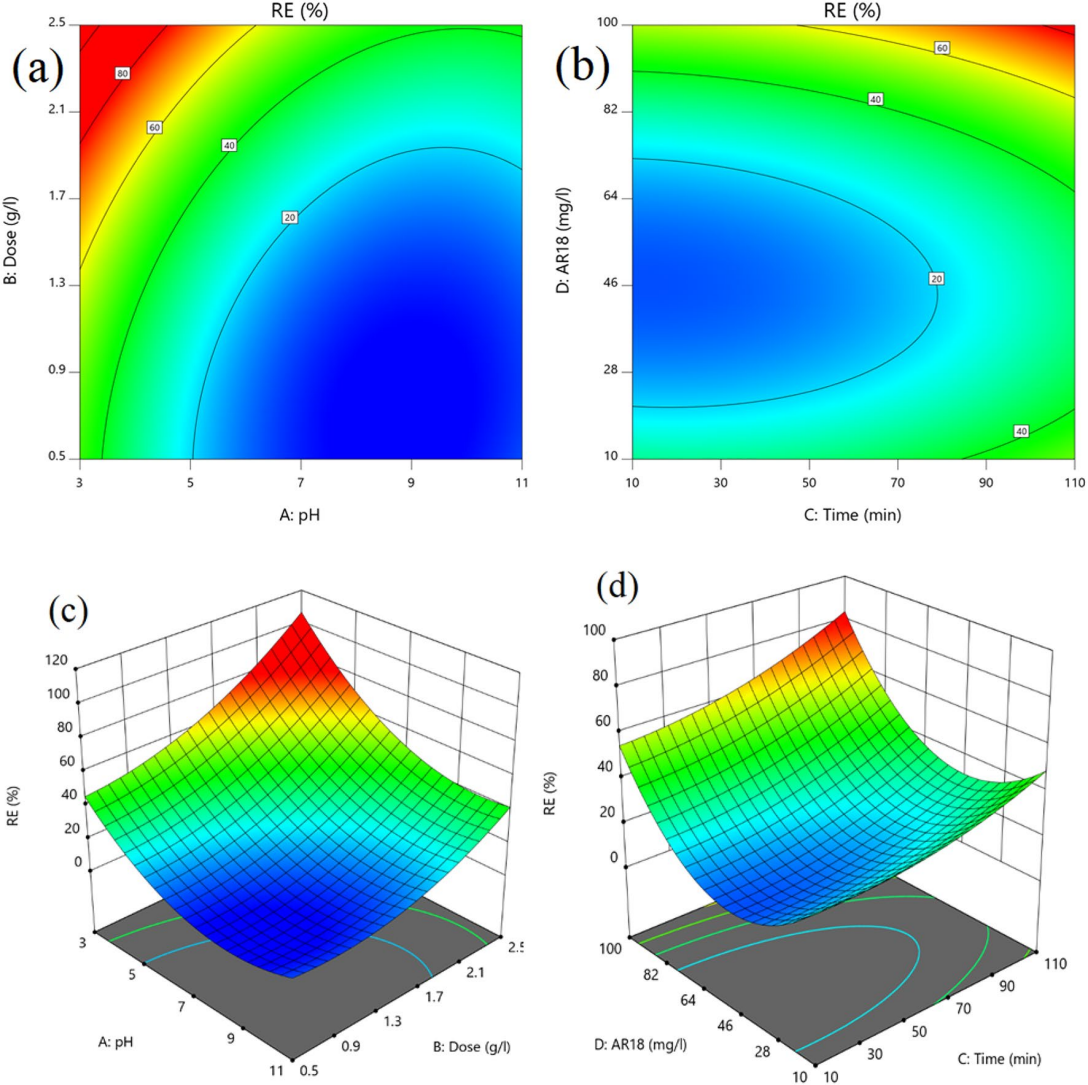
contour plots for effect of pH and adsorbent dose (**a**, **c**) and initial concentration of AR18 and time (**b**, **d**).

Contact time was found to be the less effective parameter in the adsorption process. The impact of contact time in present study was assessed from 10 to 110 min and the best removal efficiency obtained at 85 min of contact time (79%) whereas by increasing contact time from 10 to 60 min, uptake rate increased around 10%; generally, it can be said that the adsorption process of AR18 almost depends on pH and in non-acidic pH, the effect of time on the process was not found to be tangible. Figure 8 demonstrates the interaction effects of studied variables, (a,c) pH and adsorbent dose contours confirmed that decrease pH value from 11 to 3 and increase dose from 0.5 to 2.5 g/l can have a positive effect on the uptake rate of AR18, Meanwhile reducing pH value from 11 to 5 did not show any significant effect on removal efficiency. (b,d) Time and concentration contours represented the better dye removal at higher concentration and longer contact time.

### Development of regression model equation

Final Equation in Terms of Coded Factors was obtained, as shown the highest efficiency was 79.71%. The final equation shows the empirical relationship between AR18 removal (Y), based on pH (A), dosage (B), time (C) and initial dye concentration (D):

$$ \begin{aligned} {\text{AR18 removal efficiency:}}\; & {16}.{5617} - {12}.{\text{6342A}} + {11}.{\text{69B}} + {5}.{\text{91C}} + {7}.{3}0{\text{5D}} - {1}.{\text{73375AB}} \\ & \quad - {4}.{6}0{\text{25AC}} + {3}.{\text{2475AD}} - 0.{\text{5925BC}} + {3}.{\text{2125BD}} + 0.{\text{66375CD}} \\ & \quad + {5}.{\text{45125A}}^{{2}} + {3}.{\text{155B}}^{{2}} + {1}.{\text{46875C}}^{{2}} + {8}.0{\text{4125D}}^{{2}} \\ \end{aligned} $$

### Determining optimal settings

The optimum condition is the value of each variable in which the maximum uptake rate is obtained. These conditions were determined by the numerical optimization method. According to the software (RSM) the maximized efficiency expected to be 78.59% at pH = 5, adsorbent dose = 2 g/l, contact time = 85 min and initial AR18 concentration = 77.5 mg/l. The experimental RE under-designed optimum conditions was achieved 79.71%. Table 6 shows the range of prediction interval, which is 72.9–84.2%, the actual value for this investigation was 79.71%.

**Table 6:** Confidence interval of efficiency prediction.

Response	Predicted mean	Predicted median	Std Dev	95% PI low	Data mean	95% PI high
RE%	78.5896	78.5896	2.12107	72.9	79.71	84.27
Predict					78.59	

Also optimization of the process parameters was accomplished by using the GA method. In this approach, the solutions created by one population are applied to generate a new population. The generation of the new population is continued to find a better solution or fitness value. The operation is sopped as the best fitness value shows no impressive improvement by the further populations and be approximately constant. The proposed approach was performed in the Matlab GA toolbox. (Matlab2018).

**Figure 9:**
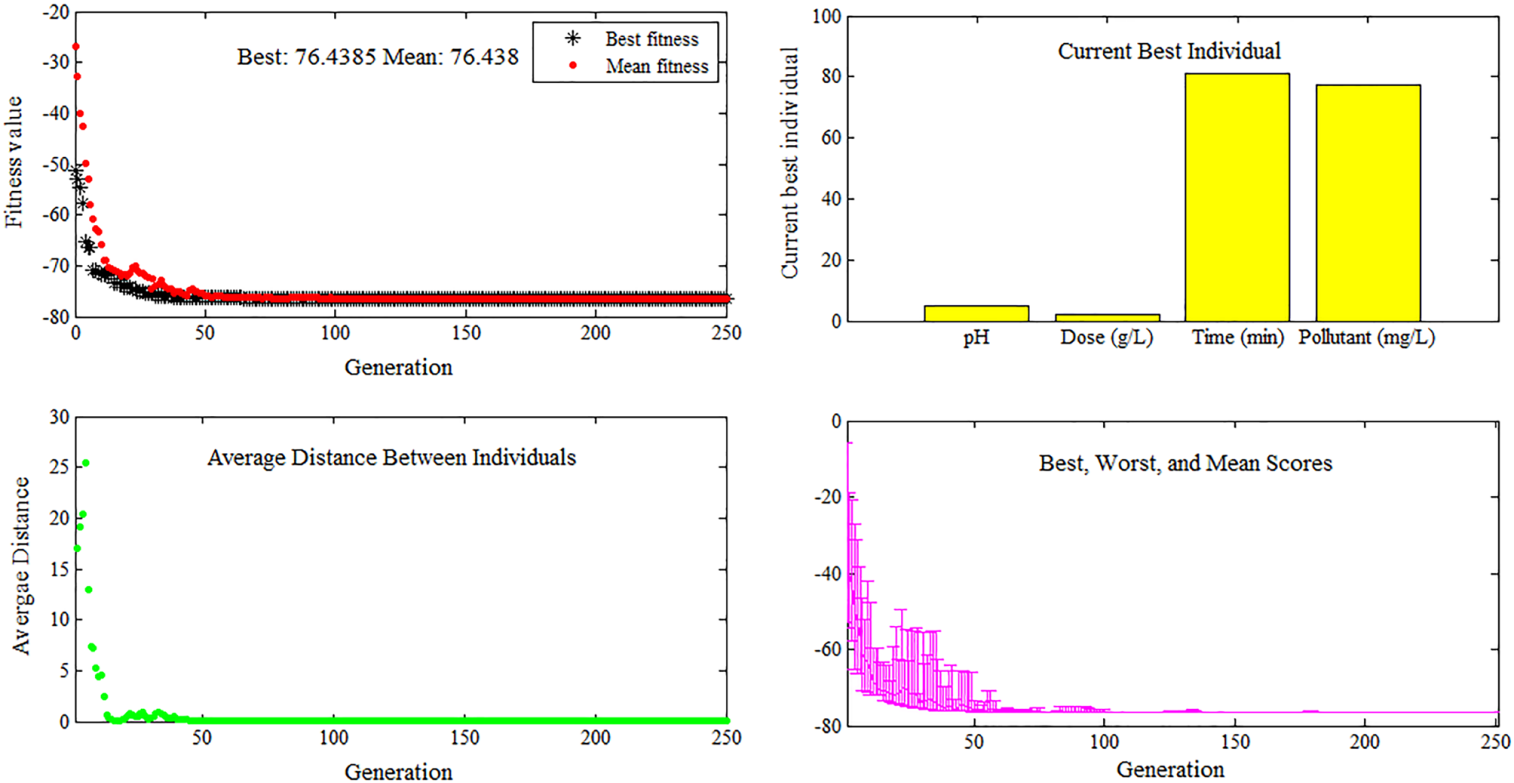
AR18 optimization using genetic algorithm.

To optimize the RSM-CCD model based on the GA approach, the minimum and maximum levels of the independent variables were set at the upper and lower levels. As seen in Fig. 9, the results evidently show that the best fitness value was improved rapidly until about generation 50, and after that, shows no impressive improvement and be approximately constant because whose populations become close to the optimal point. As given in Fig. 9, the current best individuals plot demonstrates that the maximum removal efficiency of about 76.43% is achieved at the optimum condition.

Optimum condition based on GA results obtained pH = 5, Adsorbent dose = 2 g/l, Contact time = 81 min and Dye Concentration = 77.5 mg/l. These results was found very close to RSM Optimization method.

### Isotherm studies

For better understating of adsorption mechanism, isotherm studies are essential. The equilibrium distribution of AR18 on GFH was found by isotherms, which were performed at seven different initial AR18 concentration with 2 g/l of adsorbent, at pH = 7 for 60 min of contact time at room temperature. In this study, three isotherm models were investigated included: Langmuir, Freundlich and Temkin, the model'. Also, the coefficient constants of each model are listed in the Table 7.

**Table 7:** The parameters of isotherm models for AR18 adsorption.

Adsorbent	Langmuir			Freundlich			Temkin		
	R^2^	q_max_ (mg/g)	K_L_	R^2^	K_f_ (l/mg)	n	R^2^	b_1_	k_t_
Granular ferric hydroxide	0.964	29.134	1.175	0.9844	3.152	0.687	0.891	624.99	2.41

Based on the obtained data in the Table 7, adsorption data was better fit to Freundlich Isotherm. The maximum capacity for dye adsorption was found 29.13 mg/g while the adsorption energy was 1.175 which shows that the affinity of dye molecules on GFH is not such a strong binding because a higher k_L_ value for adsorbent shows a stronger affiliation to sorbate. Also, the coefficient 1/n (generally 0–1) indicates the favorable adsorption of the adsorbate to the adsorbent.^37^ The 1/n value tends to zero; the adsorbent surface is more heterogenic^38^. The affinity of the adsorption sites between AR18 and GFH is determined by R_L_ constant, which is dimensionless. The value of R_L_ shows the nature of adsorption as follow:

**Table:** 

R_L_ value	Description
R_L_ > 1	Unfavorable
R_L_ = 1	Linear
0 < R_L_ < 1	Favorable
R_L_ = 0	Irreversible

**Figure 10:**
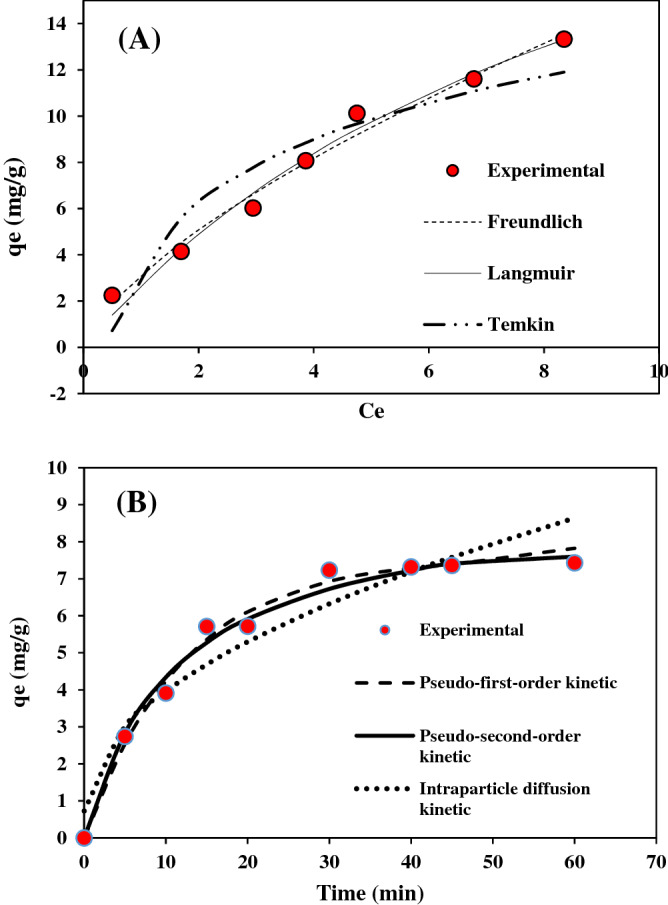
(**A**) AR18 non-linear isotherm model. (**B**) AR18 non-linear kinetic model.

The Calculated R_L_ value is between 0.14 and 0.54 as all of these values are between 0 and 1. It can understand that AR18-GFH have favorable adsorption. The non-linear fitting Isotherm models are presented in Fig. 10A.

### Kinetic studies

To understand adsorption mechanism and dye uptake rate, kinetic studies were investigated. Pseudo-first-order, Pseudo-second-order and Intra-particle diffusion models were used to analyze the adsorption kinetics. K_1_ (g/mg min), K_2_ (/min) and K_dif_ (mg/g min^1/2^) are the rate constants of pseudo-first-order, second order and Intra-particle diffusion models, respectively. In this study data of experiments was fit better with Pseudo-second-order Kinetic model (R^2^ = 0.0984) The non-linear fitting kinetic curves of these kinetic are presented in Fig. 10B. Result of Kinetics of this study is agreement with previous study^2,39,40^. The pseudo-first-order kinetic assumes that the adsorption process is affected by the physiosorption, and the rate-limiting step in adsorption depends on collision between solute molecules of ions with unoccupied single sites at the surface of the adsorbent material The intraparticle diffusion kinetic assumes that the intraparticle diffusion of dye molecules on the adsorbent is the rate-limiting step in the adsorption process^41^. The pseudo-second-order kinetic assumes that the rate-limiting step of adsorption is chemisorption, which is probably ascribed to ion-exchange or sharing of electrons between adsorbents and adsorbate. Another assumption in this model is that the rate of occupation of adsorption sites is proportional to the square of the number of unoccupied sites^42,43^. Also, the intraparticle diffusion kinetic assumes that the intraparticle diffusion of dye molecules on the adsorbent is the rate-limiting step in the adsorption process^15^. parameters of Kinetic models is shown in Table 8.

**Table 8:** The parameters of Kinetic models for AR18 adsorption.

Pseudo-first-order kinetic			Pseudo-second-order kinetic			Intraparticle diffusion kinetic		
R^2^	q_e_ (mg/g)	K_1_ (g/mg/min)	R^2^	q_e_ (mg/g)	K_2_ (/min)	K (mg/g min^1/2^)	C	R^2^
0.962	7.580	0.082	0.984	9.279	0.009	1.022	0.729	0.9216

### Thermodynamics of adsorption

Temperature is one of the factors affecting the adsorption process. Thermodynamic studies help us to understand recognize the process as much as possible, as well as information about changes in the internal energy associated with adsorption and, thus, to take measures to increase the efficiency of adsorption. In this study, the efficacy of GFH in the removal of acid red 18 at temperatures (298, 293, and 303° K) was investigated. The thermodynamic parameters at three different temperatures are listed in Table 9. The enthalpy change (**ΔH**^**0**^) obtained are Positive and demonstrates the endothermic nature the adsorption of Acid Red 18 onto GFH; this also supports the observed increase in the adsorption capacity of Acid Red 18 with increasing temperature so increasing temperature is favorable for the adsorption. The positive value of standard entropy change (ΔS) suggests stability, good affinity and decrease of randomness of Acid Red 18 by GFH in the whole removal process. The negative value of ΔG at all temperatures indicated that the adsorption was a spontaneous process. Additionally, the increase in absolute values of ΔG with increasing the temperature reveals that higher temperature facilitated the adsorption.

**Table 9:** Thermodynamic parameters of AR18 adsorption onto the GFH.

T (^o^K)	K	Ln K	ΔG (kJ/mol)	ΔH^0^ (kJ/mol)	ΔS^0^ (J/mol K)
283	2.29	0.832	− 1/95		
293	2.66	0.978	− 2/38		
303	4.05	1.399	− 3/52	20.099	0.077

In the Table 10 comparison of maximum adsorption capacity of Acid Red 18 and other adsorbents has been investigated.

**Table 10:** Comparison of maximum adsorption capacity of AR18 between various adsorbents.

	Adsorbent	pH	Isotherm	Kinetic	q_m_ (mg/g)	Refs.
1	Activated carbon walnut	5	Langmuir	PSO	30.3	^38^
2	Activated carbon poplar woods	5	Langmuir	PFO	3.91	^38^
3	Activated carbon curry tree	–	Langmuir	PFO	53.19	^44^
4	Magnetite nanoparticles	3	Freundlich	PSO	16.25	^45^
5	Sargassum Glaucescens biomass	6	Freundlich	–	15	^46^
6	Activated carbon	5	Freundlich	PFO	2	^47^
7	SMCZ	7	Langmuir	PSO	11	^48^
8	Activated carbons peach H_4_P_2_O_7_	3	Langmuir	PSO	34.24	^49^
9	mCS/CNT	3	Redlich-Peterson	PSO	771.1	^50^
10	Acidic treated pumice	3.5	Langmuir	Intra-particle	29.7	^51^
11	rGO/APT	2	Langmuir	PSO	26.59	^40^
12	Activated charcoal almond shell	2	Freundlich	PFO	10.75	^2^
13	MWCNTs	3	Langmuir	PSO	166.6	^52^
14	**Granular ferric hydroxide (GFH)**	**5**	**Freundlich**	**PSO**	**29.13**	**This study**

Significant values are in [bold/italics].

## Conclusion

In this study removal of Acid red 18 anionic dye by granular ferric hydroxide (GFH) with RSM-CCD method design with 30 runs investigated. The results showed GFH Nanocrystal can effectively reduce AR18 concentration in adsorption process (78.59%). Optimization of process performed with RSM and Genetic Algorithm method. Results of two procedure was very close together, RSM: 78.59% vs GA:76.43%. The adsorption process can be better fitted with Freundlich isotherm (R^2^ = 0.98) and Pseudo-second-order (R^2^ = 0.98) kinetic models. Maximum adsorption capacity determined 29.13 mg/g. Also thermodynamic studies indicated that the reaction process was endothermic and spontaneous. According to the obtained results, GFH can be considered as having a good efficiency in removing Acid red18 dye.

## Data Availability

All data generated or analyzed during this study are included in this published article.

## References

[1] Saleh TA, Tuzen M, Sarı A (2021). Evaluation of poly (ethylene diamine-trimesoyl chloride)-modified diatomite as efficient adsorbent for removal of rhodamine B from wastewater samples. Environ. Sci. Pollut. Res..

[2] Chaleshtori AN (2017). Removal of Acid Red 18 (Azo-Dye) from aqueous solution by adsorption onto activated charcoal prepared from almond shell. J. Environ. Sci. Manag..

[3] Saleh TA, Al-Ruwayshid SH, Sarı A, Tuzen M (2020). Synthesis of silica nanoparticles grafted with copolymer of acrylic acrylamide for ultra-removal of methylene blue from aquatic solutions. Eur. Polymer J..

[4] Acisli O, Khataee A, Karaca S, Sheydaei M (2016). Modification of nanosized natural montmorillonite for ultrasound-enhanced adsorption of Acid Red 17. Ultrason. Sonochem..

[5] Ashrafi SD, Rezaei S, Forootanfar H, Mahvi AH, Faramarzi MA (2013). The enzymatic decolorization and detoxification of synthetic dyes by the laccase from a soil-isolated ascomycete, Paraconiothyrium variabile. Int. Biodeterioration Biodegrad..

[6] Tuzen M, Sarı A, Saleh TA (2018). Response surface optimization, kinetic and thermodynamic studies for effective removal of rhodamine B by magnetic AC/CeO2 nanocomposite. J. Environ. Manage..

[7] Zoweram AN, Majlesi M, Masoudinejad MR (2016). Remove food dye (Acid Red 18) by using activated carbon of sunflower stalk modified with Iron nanoparticles Fe3O4 from aqueous solutions. Int. J. Adv. Biotechnol. Res..

[8] Altıntıg E (2021). Facile synthesis of zinc oxide nanoparticles loaded activated carbon as an eco-friendly adsorbent for ultra-removal of malachite green from water. Environ. Technol. Innovat..

[9] Gholami-Borujeni F (2011). Enzymatic treatment and detoxification of acid orange 7 from textile wastewater. Appl. Biochem. Biotechnol..

[10] Zhao T (2018). Efficient decolorization of typical azo dyes using low-frequency ultrasound in presence of carbonate and hydrogen peroxide. J. Hazard. Mater..

[11] Koupaie EH, Moghaddam MA, Hashemi S (2011). Post-treatment of anaerobically degraded azo dye Acid Red 18 using aerobic moving bed biofilm process: Enhanced removal of aromatic amines. J. Hazard. Mater..

[12] Mirzadeh S-S (2014). Decolorization of two synthetic dyes using the purified laccase of Paraconiothyrium variabile immobilized on porous silica beads. J. Environ. Health Sci. Eng..

[13] Zhang J, Liu F, Gao J, Chen Y, Hao X (2017). Ordered mesoporous TiO_2_/activated carbon for adsorption and photocatalysis of acid red 18 solution. BioResources.

[14] Bazrafshan E, Alipour MR, Mahvi AH (2016). Textile wastewater treatment by application of combined chemical coagulation, electrocoagulation, and adsorption processes. Desalin. Water Treat..

[15] Dalvand A (2016). Modeling of Reactive Blue 19 azo dye removal from colored textile wastewater using L-arginine-functionalized Fe3O4 nanoparticles: Optimization, reusability, kinetic and equilibrium studies. J. Magn. Magn. Mater..

[16] Hussain S, Khan N, Gul S, Khan S, Khan H (2019). Water Chemistry.

[17] Altintig E, Onaran M, Sarı A, Altundag H, Tuzen M (2018). Preparation, characterization and evaluation of bio-based magnetic activated carbon for effective adsorption of malachite green from aqueous solution. Mater. Chem. Phys..

[18] Ali I, Al-Hammadi SA, Saleh TA (2018). Simultaneous sorption of dyes and toxic metals from waters using synthesized titania-incorporated polyamide. J. Mol. Liquids..

[19] Altıntıg E, Altundag H, Tuzen M, Sarı A (2017). Effective removal of methylene blue from aqueous solutions using magnetic loaded activated carbon as novel adsorbent. Chem. Eng. Res. Des..

[20] Shokoohi R, Vatanpoor V, Zarrabi M, Vatani A (2010). Adsorption of Acid Red 18 (AR18) by activated carbon from poplar wood-A kinetic and equilibrium study. E-J. Chem..

[21] Bazrafshan E, Mostafapour FK, Hosseini AR, Raksh Khorshid A, Mahvi AH (2013). Decolorisation of reactive red 120 dye by using single-walled carbon nanotubes in aqueous solutions. J. Chem..

[22] Saleh TA, Elsharif AM, Bin-Dahman OA (2021). Synthesis of amine functionalization carbon nanotube-low symmetry porphyrin derivatives conjugates toward dye and metal ions removal. J. Mol. Liquids..

[23] Saha B, Bains R, Greenwood F (2005). Physicochemical characterization of granular ferric hydroxide (GFH) for arsenic (V) sorption from water. Sep. Sci. Technol..

[24] Genz A, Baumgarten B, Goernitz M, Jekel M (2008). NOM removal by adsorption onto granular ferric hydroxide: Equilibrium, kinetics, filter and regeneration studies. Water Res..

[25] Shams M, Nabizadeh Nodehi R, Hadi Dehghani M, Younesian M, Hossein Mahvia A (2010). Efficiency of granular ferric hydroxide (GFH) for removal of fluoride from water. Fluoride.

[26] Bhatnagar A (2009). Bromate removal from water by granular ferric hydroxide (GFH). J. Hazard. Mater..

[27] Lescano MR, Passalía C, Zalazar CS, Brandi RJ (2015). Arsenic sorption onto titanium dioxide, granular ferric hydroxide and activated alumina: Batch and dynamic studies. J. Environ. Sci. Health Part A.

[28] Dalvand A, Golibegloo E, Ganjali MR, Golchinpoor N, Khazaei M, Kamani H, Hosseini SS, Mahvi AH (2016). Comparison of Moringa stenopetala seed extract as a clean coagulant with Alum and Moringa stenopetala-Alum hybrid coagulant to remove direct dye from Textile Wastewater. Environ. Sci. Pollut. Res..

[29] Azari A (2019). Experimental design, modeling and mechanism of cationic dyes biosorption on to magnetic chitosan-lutaraldehyde composite. Int. J. Biol. Macromol..

[30] Moradnia M, Noorisepehr M, Salari M, Darvishmotevalli M (2021). Optimization of 2-chlorophenol removal using ultrasound/persulfate: Prediction by RSM method, biodegradability improvement of petrochemical refinery wastewater. Arab. J. Sci. Eng..

[31] Rasuli L (2021). Mesoporous metal organic frameworks functionalized with the amino acids as advanced sorbents for the removal of bacterial endotoxins from water: Optimization, regression and kinetic models. J. Mol. Liquids..

[32] Babapour M (2022). Adsorption of Cr (VI) from aqueous solution using mesoporous metal-organic framework-5 functionalized with the amino acids: Characterization, optimization, linear and nonlinear kinetic models. J. Mol. Liquids.

[33] Kyzas GZ, Peleka EN, Deliyanni EA (2013). Nanocrystalline akaganeite as adsorbent for surfactant removal from aqueous solutions. Materials.

[34] Li S (2016). Adsorptive bromate removal from aqueous solution by commercial strongly basic resin impregnated with hydrated ferric oxide (HFO): Kinetics and equilibrium studies. J. Chem. Eng. Data.

[35] Gong C, Chen D, Jiao X, Wang Q (2002). Continuous hollow α-Fe_2_O_3_ and α-Fe fibers prepared by the sol–gel method. J. Mater. Chem..

[36] Hwang S, Umar A, Dar G, Kim S, Badran R (2014). Synthesis and characterization of iron oxide nanoparticles for phenyl hydrazine sensor applications. Sens. Lett..

[37] Derakhshan Z, Baghapour MA, Ranjbar M, Faramarzian M (2013). Adsorption of methylene blue dye from aqueous solutions by modified pumice stone: Kinetics and equilibrium studies. Health Scope.

[38] Heibati B (2015). Kinetics and thermodynamics of enhanced adsorption of the dye AR 18 using activated carbons prepared from walnut and poplar woods. J. Mol. Liq..

[39] Lu F (2019). Magnetic porous polymer composite for high performance adsorption of acid red 18 based on melamine resin and chitosan. J. Mol. Liquids.

[40] Xu H (2018). Reduced graphene oxide/attapulgite-supported nanoscale zero-valent iron removal of acid red 18 from aqueous solution. Water Air Soil Pollut..

[41] Kamari S, Ghorbani F, Sanati AM (2019). Adsorptive removal of lead from aqueous solutions by amine-functionalized magMCM-41 as a low-cost nanocomposite prepared from rice husk: Modeling and optimization by response surface methodology. Sustain. Chem. Pharm..

[42] Qiao L, Du K (2021). Toluidine blue-immobilized macroporous chitosan microspheres for highly efficient purification of fucoidan. Biochem. Eng. J..

[43] Abdić Š, Memić M, Šabanović E, Sulejmanović J, Begić S (2018). Adsorptive removal of eight heavy metals from aqueous solution by unmodified and modified agricultural waste: tangerine peel. Int. J. Environ. Sci. Technol..

[44] Suresh S, Sugumar RW, Maiyalagan T (2011). Adsorption of acid red 18 from aqueous solution onto activated carbon prepared from Murraya koenigii (curry tree) seeds. Asian J. Chem..

[45] Berizi Z, Hashemi SY, Hadi M, Azari A, Mahvi AH (2016). The study of non-linear kinetics and adsorption isotherm models for Acid Red 18 from aqueous solutions by magnetite nanoparticles and magnetite nanoparticles modified by sodium alginate. Water Sci. Technol..

[46] Zazouli MA, Moradi E (2015). Adsorption Acid Red18 dye using Sargassum Glaucescens biomass from aqueous solutions. Iran. J. Health Sci..

[47] Garousin A, Bozorghi SJ, Moradi R (2015). Removal of environmental pollutants azo dye Acid Red 18 in aqueous solution using adsorbent activated carbon of walnut shell. J. biodivers. environ. sci..

[48] Mirzaei N (2017). Modified natural zeolite using ammonium quaternary based material for Acid red 18 removal from aqueous solution. J. Environ. Chem. Eng..

[49] Saratale RG (2016). Preparation of activated carbons from peach stone by H4P2O7 activation and its application for the removal of Acid Red 18 and dye containing wastewater. J. Environ. Sci. Health Part A.

[50] Wang S (2014). Highly efficient removal of acid red 18 from aqueous solution by magnetically retrievable chitosan/carbon nanotube: Batch study, isotherms, kinetics, and thermodynamics. J. Chem. Eng. Data.

[51] Samarghandi MR, Zarrabi M, Amrane A, Safari GH, Bashiri S (2012). Application of acidic treated pumice as an adsorbent for the removal of azo dye from aqueous solutions: Kinetic, equilibrium and thermodynamic studies. Iran. J. Environ. Health Sci. Eng..

[52] Shirmardi M, Mesdaghinia A, Mahvi AH, Nasseri S, Nabizadeh R (2012). Kinetics and equilibrium studies on adsorption of acid red 18 (Azo-Dye) using multiwall carbon nanotubes (MWCNTs) from aqueous solution. E-J. Chem..

